# Genetic diversity and sex‐biased dispersal in the brown spotted pitviper (*Protobothrops mucrosquamatus*): Evidence from microsatellite markers

**DOI:** 10.1002/ece3.8652

**Published:** 2022-03-01

**Authors:** Min Yu, Qin Liu, Ya‐Yong Wu, Peng Guo, Kong Yang

**Affiliations:** ^1^ 66336 Institute of Qinghai‐Tibetan Plateau Southwest Minzu University Chengdu China; ^2^ 66336 Faculty of Agriculture, Forest and Food Engineering Yibin University Yibin China

**Keywords:** genetic diversity, microsatellites, *Protobothrops mucrosquamatus*, sex‐biased dispersal, snake

## Abstract

Dispersal plays a vital role in the geographical distribution, population genetic structure, quantity dynamics, and evolution of a species. Sex‐biased dispersal is common among vertebrates and many studies have documented a tendency toward male‐biased dispersal in mammals and female‐biased dispersal in birds. However, dispersal patterns in reptiles remain poorly understood. In this study, we explored the genetic diversity and dispersal patterns of the widely distributed Asian pitviper *Protobothrops mucrosquamatus*. In total, 16 polymorphic microsatellite loci were screened in 150 snakes (48 males, 44 females, 58 samples without sex information) covering most of their distribution. Microsatellite analysis revealed high genetic diversity in *P*. *mucrosquamatus*. Bayesian clustering of population assignment identified two major clusters for all populations, somewhat inconsistent with the mitochondrial DNA phylogeny of *P*. *mucrosquamatus* reported in previous research. Analyses based on 92 sex‐determined and 37 samples of *P*. *mucrosquamatus* from three small sites in Sichuan, China (Mingshan, Yibin, and Zizhong) consistently suggested female‐biased dispersal in *P*. *mucrosquamatus*, which is the first example of this pattern in snakes. The female‐biased dispersal patterns in *P*. *mucrosquamatus* may be explained by local resource competition.

## Graphical abstract

In this study, we explored the genetic diversity and dispersal patterns of the widely distributed Asian pitviper *Protobothrops mucrosquamatus*. Microsatellite analysis revealed high genetic diversity in *P*. *mucrosquamatus*. Analyses based on sex‐determined samples suggested female‐biased dispersal in *P*. *mucrosquamatus*, which is the first example of this pattern in snakes. The female‐biased dispersal patterns in *P*. *mucrosquamatus* may be explained by local resource competition.

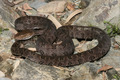

## 1 INTRODUCTION

Dispersal plays a vital role in the life history of a species by influencing population structure, quantity dynamics, genetic diversity, and species evolution (Guerrini et al., 2014; Ronce, 2007; Trochet et al., 2016). While movement may entail substantial costs in terms of death and unknown future habitat (Greenwood & Harvey, 1982; Howard, 1960), immigrant individuals gain certain benefits, such as inbreeding avoidance and increased breeding opportunities. In vertebrates, individuals of one sex often disperse more or further than individuals of the other sex, i.e., sex‐biased dispersal. Currently, 257 species have been reported to show sex‐dispersal patterns, including seven species of invertebrate arthropods, 118 species of birds, 110 species of mammals, four species of fish, 14 species of reptiles, and four species of amphibians (Trochet et al., 2016). Many studies had documented a tendency toward male‐biased dispersal in mammals and female‐biased dispersal in birds (Corrales & Höglund, 2012; Costello et al., 2008; Greenwood, 1980; Nemesházi et al., 2018; Paplinska et al., 2009; Song et al., 2015; Vangestel et al., 2013). Based on mammalian and bird studies, several hypotheses have been proposed to explain sex‐biased dispersal, including resource competition (Greenwood, 1980), local mate competition (Dobson, 1982; Perrin & Mazalov, 2000), and inbreeding avoidance (Perrin & Mazalov, 2000; Pusey, 1987). However, compared with birds and mammals, comparatively fewer studies have been conducted on dispersal patterns in reptiles (Dubey et al., 2008; Hofmann et al., 2012; Johansson et al., 2008; Keogh et al., 2007; Qi et al., 2013; Ujvari et al., 2008; Urquhart et al., 2009; Wang et al., 2019).

**FIGURE 1:**
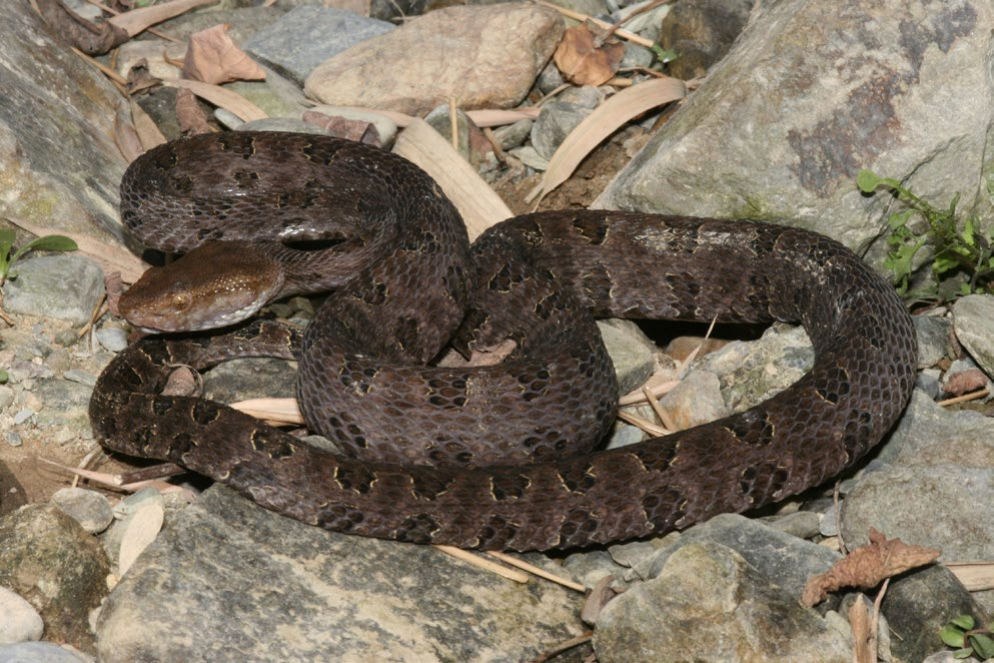
The photo of *Protobothrops mucrosquamatus* in life

*Protobothrops mucrosquamatus* (Cantor, 1839) (Figure 1) is a medium‐sized Asian pitviper distributed in southwest and southeast China, Laos, northern Bangladesh, Vietnam, northern Myanmar, and northeastern India (Zhao, 2006). Due to the wide distribution of *P*. *mucrosquamatus*, it is easy to be encountered in the field. Thus, it is a very ideal species to explore its genetics, evolution, and ecology. Zhong et al. (2017) examined and morphologically compared 142 specimens of *P*. *mucrosquamatus* and identified sexual dimorphism within the species but no significant morphological differences among the populations, despite their wide distribution. Based on two mitochondrial DNA fragments and two nuclear genes, Guo et al. (2019) explored the genetic diversity and population evolutionary history of *P*. *mucrosquamatus* and found five geographically structured and well‐supported mtDNA matrilineal lineages within the species. However, due to the limited genes, the DNA sequences did not provide much additional information on population structure.

Microsatellites, also known as simple sequence repeats (SSR), are recurring motifs of 1–6 nucleotides found in the genomes of eukaryotes (Selkoe & Toonen, 2006). In comparison to other polymerase chain reaction (PCR)‐based methods, including inter‐simple sequence repeat (ISSR), randomly amplified polymorphic DNA (RAPD), and amplified fragment length polymorphism (AFLP), microsatellites represent a powerful marker due to their codominant inheritance and high polymorphism, and have been widely used in phylogeographic, population, and parental analyses (Guichoux et al., 2011; Hodel et al., 2016; Qin et al., 2017). In this study, based on microsatellite markers, we explored the genetic diversity and population genetic structure of *P*. *mucrosquamatus*, and determined whether sex‐biased dispersal exists in this species.

## 2 MATERIALS AND METHODS

### 2.1 Sampling and RAD sequencing

**FIGURE 2:**
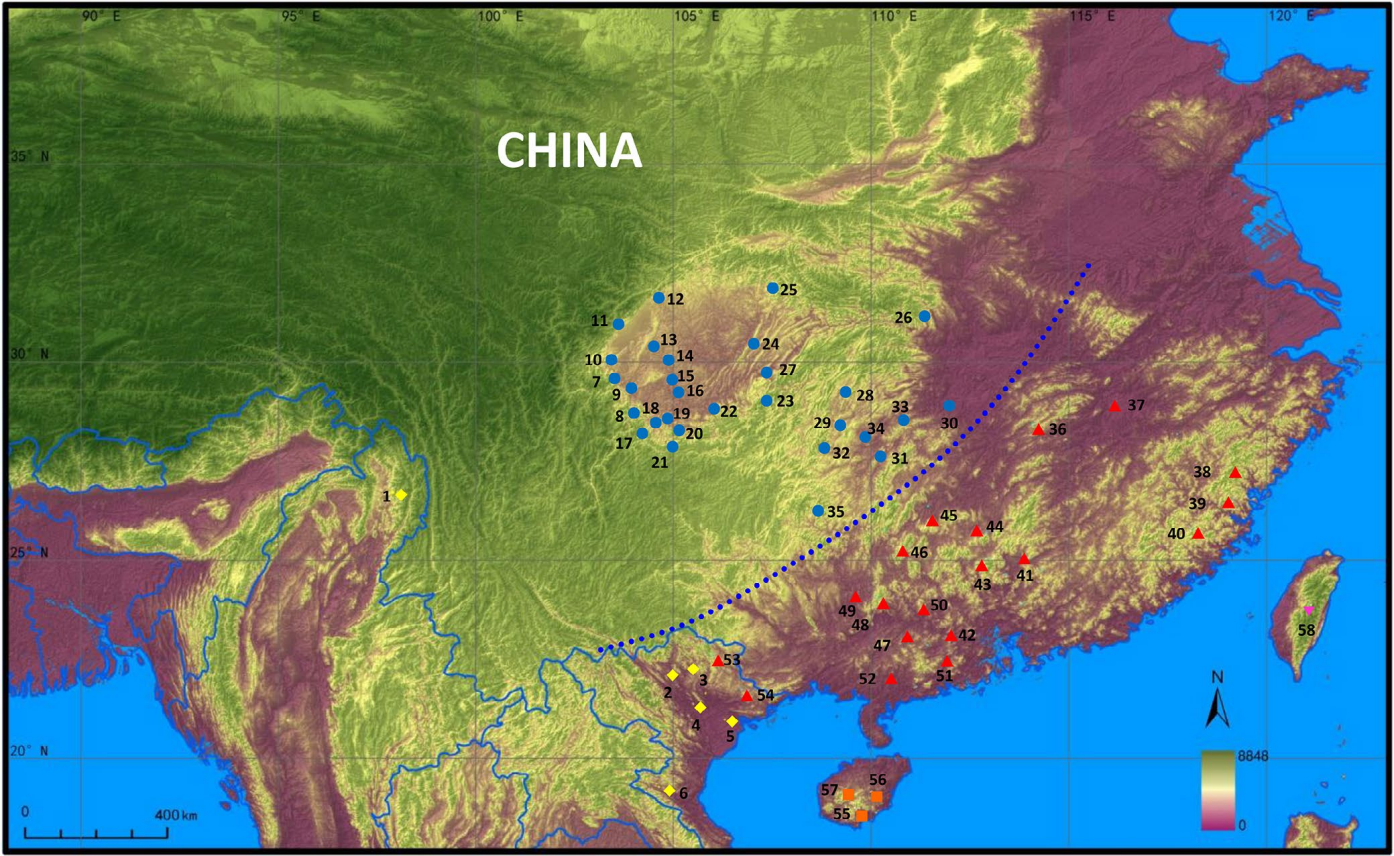
Topographic map of China and adjoining countries showing sampling localities for *Protobothrops mucrosquamatus* across 58 localities. Numbers indicate specimen localities numbered in Table 1. Blue dotted line separates two clusters detected in STRUCTURE; filled circles: SWC (blue); diamonds: VM (yellow); squares: HN (orange); inverted triangles: TW (purple); triangles: SCV (red)

In total, 150 *P*. *mucrosquamatus* snakes covering most of their range were collected between 1994 and 2018 through fieldwork or tissue loans from colleagues and museums (Figure 2 and Table 1). Liver and muscle tissue samples were taken and preserved in 90% ethanol. Whole genomic DNA was extracted using a TIANamp Genomic DNA kit (Tiangen Biotech (Beijing) Co., Ltd.) following the manufacturer's protocols.

**TABLE 1:** Sample information for *Protobothrops mucrosquamatus* analyzed in this study (CAS: California Academy of Science, San Francisco; ROM: Royal Ontario Museum, Toronto; AM: Anita Malhotra catalogue number; GP: Guo Peng, own catalogue number)

Individual ID	Location	Location No	Population	Sex
CAS224693	KaChin State, Myanmar	1	VM	
CAS232934	KaChin State, Myanmar	1	VM	
ROM6551	Tuyen Quang, Vietnam	2	VM	
ROM6809	Tuyen Quang, Vietnam	2	VM	
ROM14465	Bac Thai, Vietnam	3	VM	
AMB744	Vinh Phuc, Tam Dao, Vietnam	4	VM	
AMB746	Vinh Phuc, Tam Dao, Vietnam	4	VM	
AMB748	Vinh Phuc, Tam Dao, Vietnam	4	VM	
ROM14489	Vinh Phu, Tam Dao, Vietnam	4	VM	
ROM18207	Vinh Phu, Tam Dao, Vietnam	4	VM	
ROM24163	Hia Duong, Vietnam	5	VM	
ROM25111	Hia Duong, Vietnam	5	VM	
ROM25716	Nghe An, Vietnam	6	VM	
ROM25715	Nghe An, Vietnam	6	VM	
GP4510	Tianquan, Sichuan, China	7	SWC	
GP4682	Leshan, Sichuan, China	8	SWC	M
GP4683	Leshan, Sichuan, China	8	SWC	F
GP31	Liujiang, Hongya, Sichuan	9	SWC	
**GP2057**	**Mingshan, Sichuan, China**	**10**	**SWC**	**F**
**GP2065**	**Mingshan, Sichuan, China**	**10**	**SWC**	**M**
**GP2066**	**Mingshan, Sichuan, China**	**10**	**SWC**	**M**
**GP2068**	**Mingshan, Sichuan, China**	**10**	**SWC**	**F**
**GP2428**	**Mingshan, Sichuan, China**	**10**	**SWC**	**M**
**GP1381**	**Mingshan, Sichuan, China**	**10**	**SWC**	**M**
**GP2067**	**Mingshan, Sichuan, China**	**10**	**SWC**	**M**
GP2058	Mingshan, Sichuan, China	10	SWC	
**GP2426**	**Mingshan, Sichuan, China**	**10**	**SWC**	**M**
**GP2427**	**Mingshan, Sichuan, China**	**10**	**SWC**	**M**
**GP2422**	**Mingshan, Sichuan, China**	**10**	**SWC**	**F**
**GP2425**	**Mingshan, Sichuan, China**	**10**	**SWC**	**M**
GP2543	Dujiangyan, Sichuan, China	11	SWC	
GP1041	Anxian, Sichuan, China	12	SWC	
GP1575	Jianyang, Sichuan, China	13	SWC	M
GP314	Longquan, Sichuan, China	13	SWC	
GP1578	Jianyang, Sichuan, China	13	SWC	F
GP1579	Jianyang, Sichuan, China	13	SWC	F
GP1580	Jianyang, Sichuan, China	13	SWC	M
GP1209	Ziyang, Sichuan, China	14	SWC	M
**GP2172**	**Zizhong, Sichuan, China**	**15**	**SWC**	**F**
**GP2173**	**Zizhong, Sichuan, China**	**15**	**SWC**	**M**
**GP2174**	**Zizhong, Sichuan, China**	**15**	**SWC**	**M**
**GP2175**	**Zizhong, Sichuan, China**	**15**	**SWC**	**M**
**GP2176**	**Zizhong, Sichuan, China**	**15**	**SWC**	**M**
**GP2177**	**Zizhong, Sichuan, China**	**15**	**SWC**	**M**
**GP2178**	**Zizhong, Sichuan, China**	**15**	**SWC**	**F**
**GP2179**	**Zizhong, Sichuan, China**	**15**	**SWC**	**M**
**GP2180**	**Zizhong, Sichuan, China**	**15**	**SWC**	**F**
**GP2181**	**Zizhong, Sichuan, China**	**15**	**SWC**	**F**
**GP2182**	**Zizhong, Sichuan, China**	**15**	**SWC**	**M**
**GP2183**	**Zizhong, Sichuan, China**	**15**	**SWC**	**F**
**GP2184**	**Zizhong, Sichuan, China**	**15**	**SWC**	**F**
**GP2185**	**Zizhong, Sichuan, China**	**15**	**SWC**	**F**
GP2319	Zigong, Sichuan, China	16	SWC	F
GP2329	Zigong, Sichuan, China	16	SWC	M
GP2331	Zigong, Sichuan, China	16	SWC	M
GP2453	Pingshan, Sichuan, China	17	SWC	F
GP426	Hengjiang, Sichuan, China	18	SWC	M
GP427	Hengjiang, Sichuan, China	18	SWC	M
GP2470	Yibin, Sichuan, China	19	SWC	M
GP2669	Yibin, Sichuan, China	19	SWC	F
**GP523**	**Yibin, Sichuan, China**	**19**	**SWC**	**M**
**GP1380**	**Yibin, Sichuan, China**	**19**	**SWC**	**M**
**GP2487**	**Yibin, Sichuan, China**	**19**	**SWC**	**F**
**GP2658**	**Yibin, Sichuan, China**	**19**	**SWC**	**M**
**GP5663**	**Yibin, Sichuan, China**	**19**	**SWC**	**F**
**GP5559**	**Yibin, Sichuan, China**	**19**	**SWC**	**M**
**GP5059**	**Yibin, Sichuan, China**	**19**	**SWC**	**F**
**GP5109**	**Yibin, Sichuan, China**	**19**	**SWC**	**F**
**GP5110**	**Yibin, Sichuan, China**	**19**	**SWC**	**M**
**GP5494**	**Yibin, Sichuan, China**	**19**	**SWC**	**M**
**GP5683**	**Yibin, Sichuan, China**	**19**	**SWC**	**F**
**GP1677A**	**Yibin, Sichuan, China**	**19**	**SWC**	**M**
GP659	Changning, Sichuan, China	20	SWC	F
GP2758	junlian, Sichuan, China	21	SWC	F
GP2759	junlian, Sichuan, China	21	SWC	F
GP5342	junlian, Sichuan, China	21	SWC	
GP5355	junlian, Sichuan, China	21	SWC	
GP4368	junlian, Sichuan, China	21	SWC	F
GP4367	junlian, Sichuan, China	21	SWC	F
GP3358	junlian, Sichuan, China	21	SWC	F
GP1767	Hejiang, Sichuan, China	22	SWC	
GP965	Hejiang, Sichuan, China	22	SWC	F
GP968	Hejiang, Sichuan, China	22	SWC	F
GP1080	Nanchuang, Chongqing, China	23	SWC	F
GP2764	Guang'an, Sichuan, China	24	SWC	F
GP135	Tongjiang, Sichuan, China	25	SWC	F
GP138	Tongjiang, Sichuan, China	25	SWC	F
GP777	Yichang, Hubei, China	26	SWC	
GP849	Yichang, Hubei, China	26	SWC	
GP4726	Yidu, Hubei, China	26	SWC	
GP5107	Yichang, Hubei, China	26	SWC	M
GP4883	Beibei, Chongqing, China	27	SWC	
GP4719	Qijiang, Chongqing, China	27	SWC	
GP424	Laifeng, Hubei, China	28	SWC	
GP2001	Xiushan, Chongqing, China	29	SWC	M
GP2009	Xiushan, Chongqing, China	29	SWC	M
GP887	Taoyuan, Hunan, China	30	SWC	
GP886	Luxi, Hunan, China	31	SWC	
GP892	Luxi, Hunan, China	31	SWC	
GP2948	Jiangkou, Guizhou, China	32	SWC	
GP2968	Yinjiang, Guizhou, Sichuan	32	SWC	M
GP2976	Yinjiang, Guizhou, Sichuan	32	SWC	
GP2013	Huaihua, Hunan, China	33	SWC	M
GP4930	Guzhang, Hunan, China	34	SWC	
GP4931	Yongshun, Hunan, China	34	SWC	
GP4928	Guzhang, Hunan, China	34	SWC	
GP2012	Huaihua, Hunan, China	34	SWC	F
GP2476	Pingyang, Guizhou, China	35	SWC	F
GP2472	Pingyang, Guizhou, China	35	SWC	M
GP2916	Liuyang, Hunan, China	36	SCV	F
GP2689	Liuyang, Hunan, China	36	SCV	
GP3858	Shangrao, Jiangxi, China	37	SCV	F
GP4990	Cangnan, Zhejiang, China	38	SCV	M
GP2694	Fuzhou, Fujian, China	39	SCV	M
GP2430	Dehua, Fujian, China	40	SCV	F
GP2431	Dehua, Fujian, China	40	SCV	F
GP2217	Shixing, Guangdong, China	41	SCV	F
GP2218	Shixing, Guangdong, China	41	SCV	M
GP2040	Conghua, Guangdong, China	42	SCV	
GP2237	Conghua, Guangdong, China	42	SCV	F
GP2035	Fuzhou, Fujian, China	43	SCV	
GP749	Ruyuan, Guangdong, China	43	SCV	M
GP1585	Chenzhou, Hunan, China	44	SCV	M
GP1586	Yongzhou, Hunan, China	45	SCV	F
GP1588	Yongzhou, Hunan, China	45	SCV	M
GP1589	Yongzhou, Hunan, China	45	SCV	F
GP1590	Yongzhou, Hunan, China	45	SCV	F
GP3799	Xing'an, Guangxi, China	46	SCV	
GP3800	Xing'an, Guangxi, China	46	SCV	
GP3954	Xing'an, Guangxi, China	46	SCV	
GP3986	Xing'an, Guangxi, China	46	SCV	
GP4414	Cenxi, Guangxi, China	47	SCV	M
GP4872	Hezhou, Guangxi, China	48	SCV	F
GP745	Jinxiu, Guangxi, China	49	SCV	
GP2542	Jinxiu, Guangxi, China	49	SCV	
GP4434	Wuzhou, Guangxi, China	50	SCV	F
GP4433	Wuzhou, Guangxi, China	50	SCV	F
GP2055	Guangzhou, China	51	SCV	
GP1622	Maoming, Guangzhou, China	52	SCV	F
IEKB2492	Lang Son, Vietnam	53	SCV	
ROM26695	Cao Bang, Vietnam	54	SCV	
ROM26696	Cao Bang, Vietnam	54	SCV	
GP2121	Diaoluoshan, Hainan, China	55	HN	
AMB753	Qiongzhong, Hainan, China	56	HN	
AMB754	Qiongzhong, Hainan, China	56	HN	
GP4639	Jianfenglin, Hainan, China	57	HN	
AMA211	Taiwan, China	58	TW	
AMA231	Taiwan, China	58	TW	
AMA232	Taiwan, China	58	TW	
AMB537	Taiwan, China	58	TW	

Note

Bold represents sex‐determined individuals from the three sites from Sichuan which were used to test dispersal pattern.

High‐quality DNA was transferred to Novogene Bioinformatics Technology Co., Ltd. for restriction site‐associated DNA sequencing (RAD‐seq) according to the standard protocols, in which total genomic DNA was digested with MseI restriction enzymes. The generated library was sequenced on the Illumina HiSeq 2000 platform to produce paired‐end reads. The quality of the raw reads was assessed using FastQC v.0.11.9 (Brown et al., 2017). High‐quality reads were clustered using CD‐HIT‐EST v. 4.8.1 (Li & Godzik, 2006) and assembled into contigs using Velvet v.1.2.10 (Namiki et al., 2012).

### 2.2 Microsatellite amplification and genotyping

After quality filtering, the high‐throughput sequencing data were screened to locate tetra‐nucleotide perfect repeat microsatellite loci using MSDB v.2.4.2 software (Du et al., 2012). Primer pairs were designed using Primer v.3.0 (Untergasser et al., 2012), with amplicon size ranging from 100 to 250 bp. In total, 25 microsatellite markers were randomly selected for optimization, and 16 markers were subsequently used to evaluate the genetic diversity and dispersal patterns of *P*. *mucrosquamatus*.

### 2.3 Diversity assessment

The successfully optimized microsatellites were used to evaluate the genetic diversity of *P*. *mucrosquamatus*. PCR was performed in a 25 µl volume containing 30 ng of genomic DNA, 1 µl of each primer (10 µM), 12.5 µl of 2 × T5 Super PCR Mix (PAGE) (Beijing Tsingke Biotech Co., Ltd.), and 10 µl of nuclease‐free water. The cycling conditions included a hot start pre‐denaturation of 95°C for 4 min, followed by 35 cycles of denaturation at 94°C for 45 s, annealing at 61–63°C (according to each primer pair) for 30 s, extension at 72°C for 30 s, post‐extension at 72°C for 10 min, and heat preservation at 10°C.

The PCR product size was measured on an ABI 3730xl DNA Analyzer (Applied Biosystems) according to each forward primer labeled with fluorescent dyes (FAM, HEX, or TAMRA) and data were obtained with GeneMapper v.4.0 (Applied Biosystems). All samples were read at least three times to reduce artificial error.

All loci were characterized, and the full dataset (150 individuals) was analyzed for various genetic diversity indices. Based on the mitochondrial DNA phylogeny of *P*. *mucrosquamatus* (Guo et al., 2019), five populations were defined, i.e., Hainan (HN), Vietnam & Myanmar (VM), Southern China & Vietnam (SCV), Southwestern China (SWC), and Taiwan (TW). We used Micro‐Checker v.2.2.3 (Van Oosterhout et al., 2004) and FreeNA (Chapuis & Estoup, 2006) software to detect null alleles, stuttering, and large allele dropout errors that can occur during the interpretation of microsatellite allele sequences. If there is a higher frequency of null alleles, that is, if it exceeds 0.2 for population genetic analysis, and if it exceeds 0.08 for parental analysis, the locus can be discarded or the null allele can be eliminated by redesigning primers (Wen et al., 2013). Deviation from the Hardy‐Weinberg equilibrium (HWE) was tested for each locus across and within populations by Fisher's exact test (Guo & Thompson, 1992) implemented in GenePop v.4.6 (Rousset, 2008) using a Markov chain Monte Carlo (MCMC) approach with 10 00 steps and 1000 iterations. Cervus v.3.0 was used to calculate the number of alleles (*N*_a_), expected heterozygosity (*H*_e_), observed heterozygosity (*H*_o_), and polymorphic information content (PIC) of each microsatellite marker (Kalinowski et al., 2007). PGDSpider v.2.1.1.5 (Lischer & Excoffier, 2012) and GenAlEx v.6.5 (Peakall & Smouse, 2012) were used to perform conversions between different data formats.

### 2.4 Genetic structure

STRUCTURE v.2.3.4 (Pritchard et al., 2000) was used to infer population structure and assign individuals to subpopulations following the admixture model. What is more, we use sampling location as prior (LOCPRIOR) to assist the clustering in STRUCTURE v.2.3.4. The most likely number of genetic clusters (*K*) varied from *K* = 1 to *K* = 10, with a burn‐in of 100,000 and MCMC repeats of 1,000,000 with 10 iterations. Results were collated using Structure Harvester v.0.6.94 (Earl & Vonholdt, 2012) and visualized using Excel. Selection of the optimal *K*‐value was based on both the log‐likelihood value closest to zero and the ΔK parameter (Evanno et al., 2005). CLUMPP v.1.1.2 (Jakobsson & Rosenberg, 2007) was used to cluster repeated sampling. Distruct v.1.1 software (Rosenberg, 2004) was used to graphically display population structure. The analysis of molecular variance (AMOVA) and the coefficient of genetic differentiation among populations (*F*_st_) were performed using GenAlEx v.6.5 (Peakall & Smouse, 2012). To delineate the major ordination pattern of *P*. *mucrosquamatus* populations, a discriminant analysis of principal components (DAPC) (Jombart et al., 2010) was performed by R v3.6.1 (R Core Team, 2019) using the adegenet package (Jombart, 2008). DAPC analysis is a multivariate method used to identify and describe clusters of genetically related individuals. Genetic variation is divided into two parts: between‐group variation and within‐group variation, which maximizes the former. Linear discriminants are linear combinations of alleles that best separate clusters (Deperi et al., 2018).

### 2.5 Tests for sex‐biased dispersal

In total, 92 sex‐determined individuals (48 males, 44 females) from the SCV and SWC populations were used to evaluate sex‐biased dispersal. We assessed sex‐biased dispersal from three small sites in Sichuan (Mingshan, Yibin, and Zizhong) in China using a two‐sided test. With reference to Goudet (1995), Goudet et al.’s (2002), Johansson et al.’s (2008), Hofmann et al.’s (2012), and Wang et al.’s (2019) studies on sex‐biased dispersal, we choose six parameters to evaluate the sex‐biased dispersal pattern of the *P*. *mucrosquamatus*. We calculated *F*_st_ (Hartl & Clarck, 1997), *F*_is_, genetic diversity (*H*_s_), relatedness (*r*), mean assignment index (mAIc) (Favre et al., 1997), and variance of assignment index (vAIc) for each sex separately using FSTAT v.1.2. (Goudet, 1995). Statistical significance for these indices was determined by 10,000 randomizations. We chose the unbiased Weir and Cockerham estimator to calculate *F*_st_ across all populations (Weir & Cockerham, 1984), with values generally higher for the philopatric sex than the dispersing sex. *F*_is_ describes how well genotype frequencies within populations fit the HWE, with values generally higher for the dispersing sex than the philopatric sex. Within‐group Hs values are also higher for the group with the greatest dispersal. In the case of sex‐biased dispersal, mAIc values should be lower for the dispersing sex than for the philopatric sex (Lampert et al., 2003). In contrast, vAIc values should be higher for the dispersing sex because members will include both residents (with common genotypes; positive values) and immigrants (with rare genotypes; negative values). In brief, higher *F*_is_, Hs, and vAIc values and lower *F*_st_, mAIc, and *r* values tend to be found in the dispersing sex than in the philopatric sex (Wang et al., 2019).

To further verify the results of sex‐biased dispersal, we analyze data from the 92 sex‐determined individuals and three small sites separately, we calculated and compared relatedness values between the sexes using COANCESTRY v.1.0 with five moment and two likelihood estimators (Wang, 2011).

## 3 RESULTS

### 3.1 Genetic diversity

Based on genotyping of 25 microsatellites in 150 *P*. *mucrosquamatus* individuals, 16 microsatellites were successfully optimized with polymorphic and call rates above 90% across all samples. Statistics calculated for the 16 polymorphic microsatellite loci across the sampling localities are listed in Table 2. There was no evidence of scoring error due to stuttering, and no large allele dropout was observed for any of the loci. Null alleles accounted for a certain percentage within HN, SWC, and TW populations (see Appendix S1). The null allele frequency results showed that only YM‐17 loci in HN and TW population exceeded 0.2. It may be that there are some missing sites in these two populations, but the null alleles frequency in the other three populations does not exceed 0.2. Thus, we retained this locus. What is more, the results of the Hardy‐Weinberg Equilibrium test show that some populations have 2–6 microsatellite sites deviation from the Hardy‐Weinberg, while the populations HN and TW have no loci deviate from the Hardy‐Weinberg (Appendix S2). This may be related to the widespread distribution of this species.

**TABLE 2:** Sixteen microsatellite loci information

Loci	Primer sequence (5’−3’)	Repeat motif	Size range (bp)	Tm (°C)	Labelling dye
YM‐1	F:ATAGATGGTGGAAGGAAGGAAAG	(GAAA)9	112–208	62	FAM
	R:CTCAGGGTGTCCTGTTTATTGAG				
YM‐2	F:ATATTGTTTAGGCCTCCCTGAAG	(ATGA)9	116–192	62	HEX
	R:CACATTTTGCCTCAACCACTTAT				
YM‐3	F:ACTGTTAAACCACCCAGAGTCAA	(TGAA)8	102–188	63	TAMRA
	R:TAATTCAGGAGATTGTAGCCCAA				
YM‐4	F:ATTCGTGGTTTTTAGTATCGCCT	(AATA)8	116–200	62	FAM
	R:GGAAATTTTTCCTGATTTCCAAC				
YM‐5	F:CATTCAAAGCATCCATTTTAACC	(GGAA)8	118–236	62	HEX
	R:TTCTGCTGCTCTTAAATTCCTTG				
YM‐8	F:AACCCAGGATAGGAAAGTGGTTA	(ATTC)8	114–190	62	FAM
	R:ATTGTCTGGGAAAGGAGATTGAT				
YM‐11	F:AAATCCTGTTCTTTCACCAAAAA	(ATAG)8	86–266	61	TAMRA
	R:AGTTTCTAAAGCCATGGTGAGAT				
YM‐12	F:TACATGGAAAGAGGGGTAATGAA	(TCAT)8	99–207	61	FAM
	R:CAGAAGAAAAGGTTTGACATTGG				
YM‐13	F:GGGCCTTGTATCAACTAACACAG	(TTAT)8	100–188	63	HEX
	R:AGAGTTACAATGGGCAGCAAATA				
YM‐15	F:GGTAGCTGCTCAGAGTTTGGTAA	(AGGA)8	142–211	63	TAMRA
	R:ATTGTGTAGCAGGCAGCTCTAGT				
YM‐17	F:TATTGTTGAAAACCATTCCCTCA	(TATG)8	100–198	63	FAM
	R:GGATCCAATCCTGTAGGAAAAAT				
YM‐18	F:GTATGCTGCTCAGAGTCCCCTA	(ATGA)8	144–204	63	HEX
	R:ACTGCCTTGCTGACAATCTTTT				
YM‐20	F:CTTTTGAGAGCAAGCAACAAAAT	(GTCT)8	170–238	63	TAMRA
	R:AAATGGTGTCCACAACTTGAGAT				
YM‐21	F:CATGACCTGAAAAGTCAGCATTT	(AAGA)8	118–240	62	FAM
	R:ATGTCCTTGCATTGGTTCATATC				
YM‐22	F:TGCATCCTGTTAGTCACAAAAGA	(AAAC)8	104–168	62	HEX
	R:GCAAACATTAAAACAAGCACACA				
YM‐23	F:ACAAATTCTGGTTTCAGCACATC	(TGAA)8	116–208	62	TAMRA
	R:AAATTCATGTTGTCCAAAGTTGC				

The overall level of polymorphism detected in the 16 loci was high, with total alleles of 364 and average number of alleles (Na) of 22.75 (ranging from 13 to 37). Ho varied from 0.480 (YM‐3) to 0.899 (YM‐20), with an average of 0.764. The highest He value was 0.951 (YM‐11) (average 0.891). The highest PIC value was 0.945 (YM‐11) (average 0.879). Statistics for the 16 polymorphic microsatellite loci for total dataset are listed in Table 3.

**TABLE 3:** Summary statistics for 16 polymorphic microsatellite loci overall the sampling localities (*N* = 150). The mean number of samples analyzed (*N*), a number of alleles identified (*N*_a_), observed heterozygosity (*H*_o_), expected heterozygosity (*H*_e_), Polymorphic Information Content (PIC)

Locus	*N*	*N*_a_	*H*_o_	*H*_e_	PIC
YM‐1	146	23	0.753	0.939	0.932
YM‐2	148	18	0.804	0.904	0.892
YM‐3	150	22	0.480	0.837	0.824
YM‐4	150	18	0.700	0.792	0.776
YM‐5	149	29	0.805	0.935	0.928
YM‐8	148	20	0.743	0.900	0.888
YM‐11	143	37	0.874	0.951	0.945
YM‐12	140	23	0.85	0.882	0.867
YM‐13	139	24	0.885	0.937	0.930
YM‐15	145	21	0.793	0.899	0.887
YM‐17	143	21	0.629	0.913	0.903
YM‐18	147	17	0.755	0.887	0.874
YM‐20	149	29	0.899	0.938	0.931
YM‐21	149	25	0.859	0.929	0.921
YM‐22	146	13	0.678	0.713	0.671
YM‐23	144	24	0.729	0.909	0.899
Average	146	22.75	0.764	0.891	0.879

### 3.2 Population genetic structure

**FIGURE 3:**
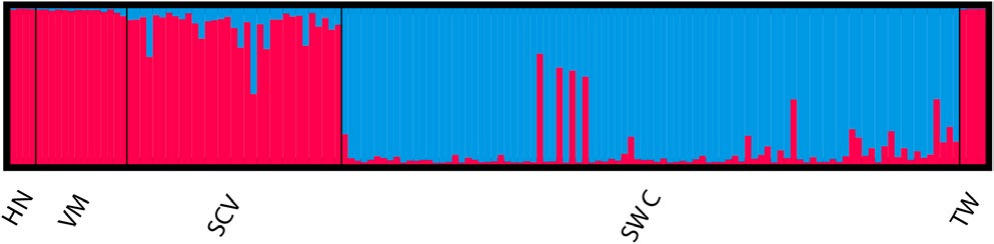
Structure diagram generated by STRUCTURE according to *K* = 2

**FIGURE 4:**
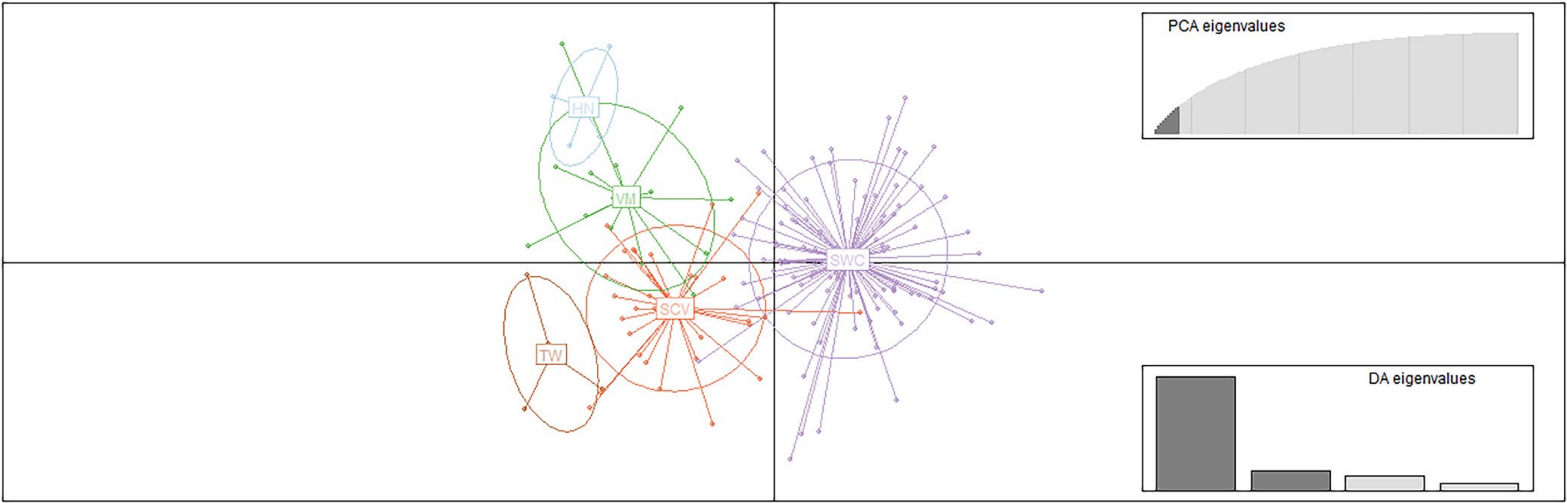
Scatter plot of the first and second principal coordinates based on the discriminant analysis of principal components (DAPC) of SSR markers. The axes represent the first two Linear Discriminants (LD). Each circle represents a cluster and each dot represents an individual. Letters represent the different populations identified by DAPC analysis

To analyze the genetic structure of *P*. *mucrosquamatus* populations, the coancestry relations of the populations were analyzed based on a Bayesian clustering model. The independent clustering of all samples recorded the highest Δ*K* value at *K* = 2 (Evanno et al., 2005), thus supporting the presence of two clusters (Appendix S3). The STRUCTURE bar plot also supported two genetic clusters (Figure 3). When *K* was 2, the genetic information of 150 samples from 5 populations came from two differential ancestral populations. At *K* = 2, most of the genetic information of 4 populations (HN, VM, SCV, and TW) in southern China and Myanmar Vietnam came from the same ancestral population (blue), while 1 population in southwestern China (SWC), the genetic information is mainly from another ancestral group (red). The two clusters displayed different population membership to that reported previously based on mtDNA (Guo et al., 2019), but were consistent with geographical origin. From the bar plot of various *K* values (*K *= 2–6), the majority of individuals revealed low probabilities of being assigned to any particular clusters (Appendix S4). DAPC analysis was carried out using the detected number of clusters (Figure 4). In Figure 4, Linear Discriminant 1 (LD 1) separated among the *P*. *mucrosquamatus* species (cluster 1 = HN, VM, SCV, TW populations, cluster 2 = SWC population) and Linear Discriminant 2 (LD 2) separated among *P*. *mucrosquamatus* cluster (HN, VM, SCV, TW populations). SWC population were roughly at the same level with respect to LD 2, and HN, VM and SCV, TW populations were above and below them, respectively. AMOVA of the five populations showed that 82% of the variation was found among individuals, with only 4% found among populations (see Appendix S5). The coefficient of genetic differentiation among populations (*F*_st_) was high in HN, VM, SCV, and SWC populations compared to the TW population. *F*_st_ values between VM and SCV, SWC populations, and SCW with SWC population were low, suggesting low genetic differentiation among them (Appendix S6).

### 3.3 Sex‐biased dispersal in *Protobothrops mucrosquamatus*

For the 92 individuals, females had higher *F*_is_ (female: 0.1662, male: 0.0831), *H*_s_ (female: 0.8770, male: 0.8597), and vAIc values (female: 64.0346, male: 35.2241) compared to males, but lower *F*_st_, mAIc, and *r* values (Table 4). However, most indices did not reveal statistical significance. Analyses from the three sites (Mingshan, Yibin, and Zizhong) showed that females had higher *F*_is_ (0.1113 vs. 0.0347), *H*_s_ (0.8174 vs. 0.7907), and vAIc values (14.6314 vs. 12.5667) compared to males, but lower *F*_st_, mAIc, and *r* values (Table 5). When we examined the three sites separately, two out of seven relatedness indices were significantly higher in males than in females (*p* < .05) (Table 6).

**TABLE 4:** Genetic differentiation (*F*_st_), inbreeding coefficient (*F*_is_), within‐site gene diversity (*H*_s_), relatedness (*r*), mean assignment index (mAIc), and variance of assignment index (vAIc) for the 92 individuals for females (*F*) and males (M) of *P*. *mucrosquamatus*

	*F*_st_	*F*_is_	*H*_s_	mAIc	vAIc	*r*
F	0.0273	0.1662	0.8770	−1.1706	64.0346	.0460
M	0.0321	0.0831	0.8597	1.2771	35.2241	.0577
*P* value	.7393	.0012	.1052	.0975	.0785	.6250

Note

*p* Values are from two‐sided tests.

**TABLE 5:** Genetic differentiation (*F*_st_), inbreeding coefficient (*F*_is_), within‐site gene diversity (*H*_s_), relatedness (*r*), mean assignment index (mAIc), and variance of assignment index (vAIc) for the three sites in Sichuan, China for females (F) and males (M) of *P*. *mucrosquamatus*

	*F*_st_	*F*_is_	*H*_s_	mAIc	vAIc	*r*
F	0.0601	0.1113	0.8174	−1.6936	14.6314	.1033
M	0.0817	0.0347	0.7907	1.1547	12.5667	.1467
*P* value	.2117	.0711	.1699	.0379	.7775	.1253

Note

*p* Values are from two‐sided tests.

**TABLE 6:** The relatedness of females and males in 92 individuals and three sites separately

Population	Gender	Seven estimators						
		TrioML	*Wang*	*LynchLi*	LynchRd	Ritland	QuellerGt	DyadML
92 individuals	Females	0.0458	−0.03446	−0.02470	−0.02214	−0.025	−0.02171	0
	Males	0.0412	−0.02291	−0.01674	−0.02418	−0.0254	−0.02297	0
Three sites	Females	0.03042	−0.04087	−0.03680	−0.07187	−0.07642	−0.07153	0
	Males	0.03814	−0.02410	−0.02487	−0.04764	−0.04970	−0.04786	0
Mingshan	Females	0.00000	−0.01150	−0.00177	−0.50003	−0.40330	−0.49903	0
	Males	0.00706	−0.00674	−0.02015	−0.14285	−0.13675	−0.14303	0
Zizhong	Females	0.0225	−0.01803	−0.04474	−0.16841	−0.1673	−0.16995	0
	Males	0.016	−0.00292	−0.02008	−0.16666	−0.1636	−0.16759	0
Yibin	Females	0.001	−0.07384	−0.08568	−0.25147	−0.2515	−0.25377	0
	Males	0.0027	−0.00468	−0.02410	−0.16736	−0.1575	−0.16912	0

Note

Italic means *p *< .05.

## 4 DISCUSSION

### 4.1 Genetic diversity and population structure

Microsatellite markers represent a powerful tool for determining the genetic diversity of populations and are widely used in vertebrate studies (e.g., *Aipysurus laevis*, *Thermophis bailey*, *Leptobrachium boringii*) (Hofmann et al., 2012; Lukoschek et al., 2008; Wang et al., 2019). Our research showed that these markers were detected at high levels of genetic variation within *P*. *mucrosquamatus*, with multiple alleles (*N*_a_ = 22.75), high *H*_o_ (0.480–0.899), and high *H*_e_ (0.713–0.951) (Table 3). These results are consistent with previous findings based on mtDNA (Guo et al., 2019), but are higher than that detected using microsatellite markers in smooth snakes (*Coronella austriaca*) (*H*_o_ = 0.357–0.507, *H*_e_ = 0.418–0.601) (Pernetta et al., 2011) and olive sea snakes (*Aipysurus laevis*) (*H*_o_ = 0.222–0.847, *H*_e_ = 0.263–0.881) (Lukoschek et al., 2008) and comparable to that reported in slatey‐grey snakes (*Stegonotus cucullatus*) (*H*_o_ = 0.62–0.84, *H*_e_ = 0.55–0.83) (Dubey et al., 2008). In addition, the mean PIC (0.879) of *P*. *mucrosquamatus* was >0.5, indicating that this species was highly genetically diverse. Higher genetic diversity could be attributed to their wide regional distribution and varied habitats.

Based on genetic structure analysis, we detected two clusters in *P*. *mucrosquamatus*, different from previous mtDNA‐based findings (Guo et al., 2019) to some extent. This difference may be due to different genetic and evolutionary patterns between mtDNA and microsatellite markers. However, these two clusters displayed significant admixture, consistent with AMOVA results, which indicated variation among individuals (Appendix S5). A standard AMOVA for the 5 populations (without a hierarchy of regions) showed that 82% of the variation was located between individuals and only 4% among populations. In China, the last global glaciation, termed the Dali glaciation (DLG), occurred during 0.07–0.01 Ma (Shi & Wang, 1979). In Guo et al. (2019), three lines of evidence suggested that all defined matrilineal lineages of *P*. *mucrosquamatus* have experienced recent population expansion. The expansion of TW and VM populations was estimated to occurred about 0.03–0.04 Ma, which was close to the mid‐DLG, while the SWC population experienced a rapid expansion after the DLG (~0.005 Ma) when the temperature rose (Shi & Wang, 1979). However, the SCV population experienced an expansion before 0.07 Ma, which may have been triggered by pre‐Glacial Maximum. High temperatures.

### 4.2 Sex‐biased dispersal

In general, the *F*_is_, *F*_st_, *r*, mAIc, vAIc, and Hs parameters are indicative of sex‐biased dispersal patterns. Previous studies have shown that *F*_st_ is higher for the more philopatric sex than for the more dispersing sex (Goudet et al., 2002). Members of the dispersing sex also display higher *F*_is_ than the philopatric sex. Furthermore, Hs is generally higher in the group showing greater dispersal. In the case of sex‐biased dispersal, mAIc values are lower for the dispersing sex than for the philopatric sex (Lampert et al., 2003); in contrast, vAIc values are higher for the dispersing sex because members will include both residents and immigrants. Based on our total dataset, females had higher *F*_is_, *H*_s_, and vAIc values, but lower *F*_st_, *r*, and mAIc values than males (Tables 4 and 5), suggesting that *P*. *mucrosquamatus* snakes exhibit female‐biased dispersal. This result differs from previous studies on sex‐biased dispersal in snakes (e.g., *Stegonotus cucullatus*, *Drymarchon couperi*, *Thermophis baileyi*, *Rhinoplocephalus nigrescens*, *Aipysurus laevis*, *Coronella austriaca*, and *Vipera aspis*) (Dubey et al., 2008; Folt et al., 2019; Hofmann et al., 2012; Keogh et al., 2007; Lukoschek et al., 2008; Pernetta et al., 2011; Zwahlen et al., 2021). However, most indices representing sex‐biased dispersal did not differ significantly, which may be the result of incomplete sampling. Several hypotheses have been proposed for female‐dispersal in birds and mammals, including local resource competition (Greenwood, 1980), local mate competition (Dobson, 1982; Perrin & Mazalov, 2000; Rivas & Burghardt, 2005), and inbreeding avoidance (Perrin & Mazalov, 2000; Pusey, 1987). Although the true mechanism of sex‐biased dispersal is unknown in this species, we hypothesize local resource competition may better explain the dispersal pattern as females need to acquire more resources while avoiding increased competition for resources. *P*. *mucrosquamatus* is widely distributed in southeastern and southwestern China, Laos, Bangladesh, northern Vietnam, northern Myanmar, and northeastern India. It is one of the most widely distributed members in this genus, and its distribution covers different climates and vegetation types (Zhao, 2006). Maybe it has something to do with the females of this species being more inclined to dispersal.

## CONFLICTS OF INTEREST

The authors declare no conflicts of interest.

## AUTHOR CONTRIBUTIONS

**Min Yu:** Conceptualization (lead); Data curation (lead); Formal analysis (supporting); Methodology (equal); Validation (equal); Visualization (supporting); Writing – original draft (equal); Writing – review & editing (equal). **Qin Liu:** Conceptualization (supporting); Data curation (supporting); Formal analysis (lead); Investigation (equal); Methodology (equal); Software (lead); Visualization (equal); Writing – review & editing (equal). **Ya‐yong Wu:** Conceptualization (supporting); Data curation (supporting); Formal analysis (lead); Investigation (equal). **Peng Guo:** Conceptualization (lead); Data curation (lead); Formal analysis (supporting); Funding acquisition (supporting); Investigation (supporting); Methodology (supporting); Project administration (lead); Visualization (supporting); Writing – original draft (equal); Writing – review & editing (supporting). **Kong Yang:** Formal analysis (supporting); Investigation (supporting); Methodology (supporting); Software (supporting); Visualization (supporting); Writing – original draft (equal); Writing – review & editing (supporting).

## Supporting information

Appendix S1

Appendix S2

Appendix S3

Appendix S4

Appendix S5

Appendix S6

## Data Availability

All microsatellite genotypes for all individuals are deposited in Dryad https://datadryad.org/stash/share/Ntrk9UMZIhu7Zag5DOv0c8d1yXIsF8Fd2BJzgGtE4WA. All genetic analyses were performed with publicly available programs.
