# Supplementary material for: Genetic diversity and sex‐biased dispersal in the brown spotted pitviper (Protobothrops mucrosquamatus): Evidence from microsatellite markers
Source: Ecol Evol. 2022 Mar 1;12(3):e8652. doi: 10.1002/ece3.8652 (PMC8888261; doi:10.1002/ece3.8652)
Supplement: Supplementary file 1 — Appendix S1 [file ECE3-12-e8652-s002.docx]

**APPENDIX 1**  Estimation of the frequency of null alleles in the populations.

| Locus | Pop HN | Pop VM | Pop SCV | Pop SWC | Pop TW |
| --- | --- | --- | --- | --- | --- |
| YM-1 | 0 | 0.0003 | 0.09236 | 0.09861 | 0.0005 |
| YM-2 | 0 | 0 | 0.044 | 0.02951 | 0 |
| YM-3 | 0.0005 | 0.13023 | 0.07576 | **0.22529** | 0.19665 |
| YM-4 | 0.001 | 0.03067 | 0.0214 | 0.00032 | 0.10733 |
| YM-5 | 0 | 0.01921 | 0.08529 | 0.03393 | 0 |
| YM-8 | 0.0005 | 0 | 0.0623 | 0.07986 | 0 |
| YM-11 | 0 | 0.00027 | 0.06782 | 0.03338 | 0 |
| YM-12 | 0 | 0 | 0.05409 | 0.00315 | 0 |
| YM-13 | 0 | 0 | 0.05365 | 0.02091 | 0 |
| YM-15 | 0 | 0.01974 | 0.05723 | 0.04181 | 0 |
| YM-17 | **0.33333** | 0.11505 | 0.13895 | 0.09818 | **0.29029** |
| YM-18 | 0.16667 | 0.0491 | 0.04732 | 0.04109 | 0.04731 |
| YM-20 | 0 | 0.14821 | 0.00001 | 0.00673 | 0.0005 |
| YM-21 | 0.16667 | 0 | 0 | 0.04102 | 0.0005 |
| YM-22 | 0 | 0.08848 | 0 | 0.00143 | 0.001 |
| YM-23 | 0.13173 | 0.09102 | 0.0597 | 0.05873 | 0.13173 |
| Mean | 0.05 | 0.04326 | 0.05374 | 0.05087 | 0.04848 |

*Bold font represents p-value greater than 0.2.
