# Supplementary material for: Genetic diversity and sex‐biased dispersal in the brown spotted pitviper (Protobothrops mucrosquamatus): Evidence from microsatellite markers
Source: Ecol Evol. 2022 Mar 1;12(3):e8652. doi: 10.1002/ece3.8652 (PMC8888261; doi:10.1002/ece3.8652)
Supplement: Supplementary file 2 — Appendix S2 [file ECE3-12-e8652-s001.docx]

**APPENDIX 2** Results of Hardy-Weinberg disequilibrium test

| Locus | Pop HN | Pop VM | Pop SCV | Pop SWC | Pop TW |
| --- | --- | --- | --- | --- | --- |
| YM-1 | 1.0000 | 0.6315 | 0.0359 | **0.0000*** | 0.4535 |
| YM-2 | 1.0000 | 0.7822 | 0.0685 | 0.5765 | 1.0000 |
| YM-3 | 0.3248 | 0.1017 | **0.0000*** | **0.0000*** | 0.1451 |
| YM-4 | Na | 0.0420 | 0.0360 | 0.1457 | 0.3179 |
| YM-5 | 0.7689 | 0.4016 | **0.0000*** | 0.0221 | 1.0000 |
| YM-8 | 0.3175 | 0.7400 | 0.0262 | 0.0220 | 0.7583 |
| YM-11 | 1.0000 | 0.5759 | 0.0069 | **0.0004*** | 0.4217 |
| YM-12 | 1.0000 | 0.9830 | 0.0500 | 0.0798 | 0.4446 |
| YM-13 | 1.0000 | 0.6266 | **0.0000*** | 0.2392 | 1.0000 |
| YM-15 | 1.0000 | 0.2716 | 0.0216 | 0.0040 | 1.0000 |
| YM-17 | 0.3354 | 0.0287 | **0.0000*** | **0.0000*** | 0.1401 |
| YM-18 | 0.0355 | 0.3242 | 0.0043 | 0.3079 | 1.0000 |
| YM-20 | 1.0000 | **0.0000*** | 0.5273 | 0.4630 | 0.3359 |
| YM-21 | 0.0236 | 0.5314 | 0.1569 | **0.0003*** | 0.3147 |
| YM-22 | 0.3128 | 0.1877 | 0.3376 | 0.0484 | Na |
| YM-23 | 0.0840 | **0.0009*** | 0.0162 | **0.0000*** | 0.0648 |

* Indicates a significant deviation from the Hardy-Weinberg.
