# Supplementary figures and images for: Genetic diversity and sex‐biased dispersal in the brown spotted pitviper (Protobothrops mucrosquamatus): Evidence from microsatellite markers
Source: Ecol Evol. 2022 Mar 1;12(3):e8652. doi: 10.1002/ece3.8652 (PMC8888261; doi:10.1002/ece3.8652)

**APPENDIX 3** Estimation of amount of K (population structure)


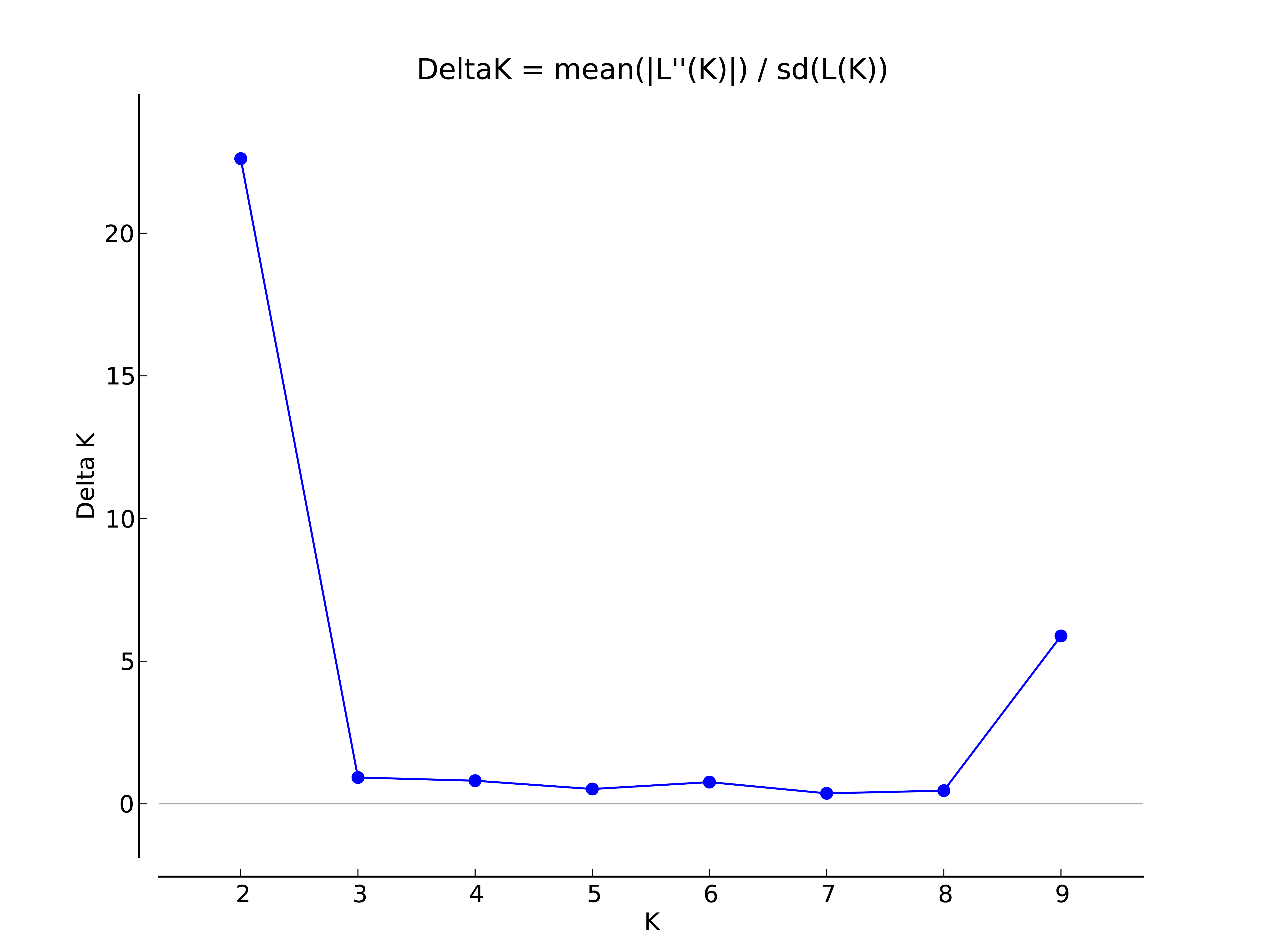

Supplement: Supplementary file 3 — Appendix S3 [file ECE3-12-e8652-s003.docx]
