# Supplementary material for: Genetic diversity and sex‐biased dispersal in the brown spotted pitviper (Protobothrops mucrosquamatus): Evidence from microsatellite markers
Source: Ecol Evol. 2022 Mar 1;12(3):e8652. doi: 10.1002/ece3.8652 (PMC8888261; doi:10.1002/ece3.8652)

**APPENDIX 4** Bar plot of the individual assignment results. Each vertical bar represents

one individual and its probabilities of being assigned to cluster


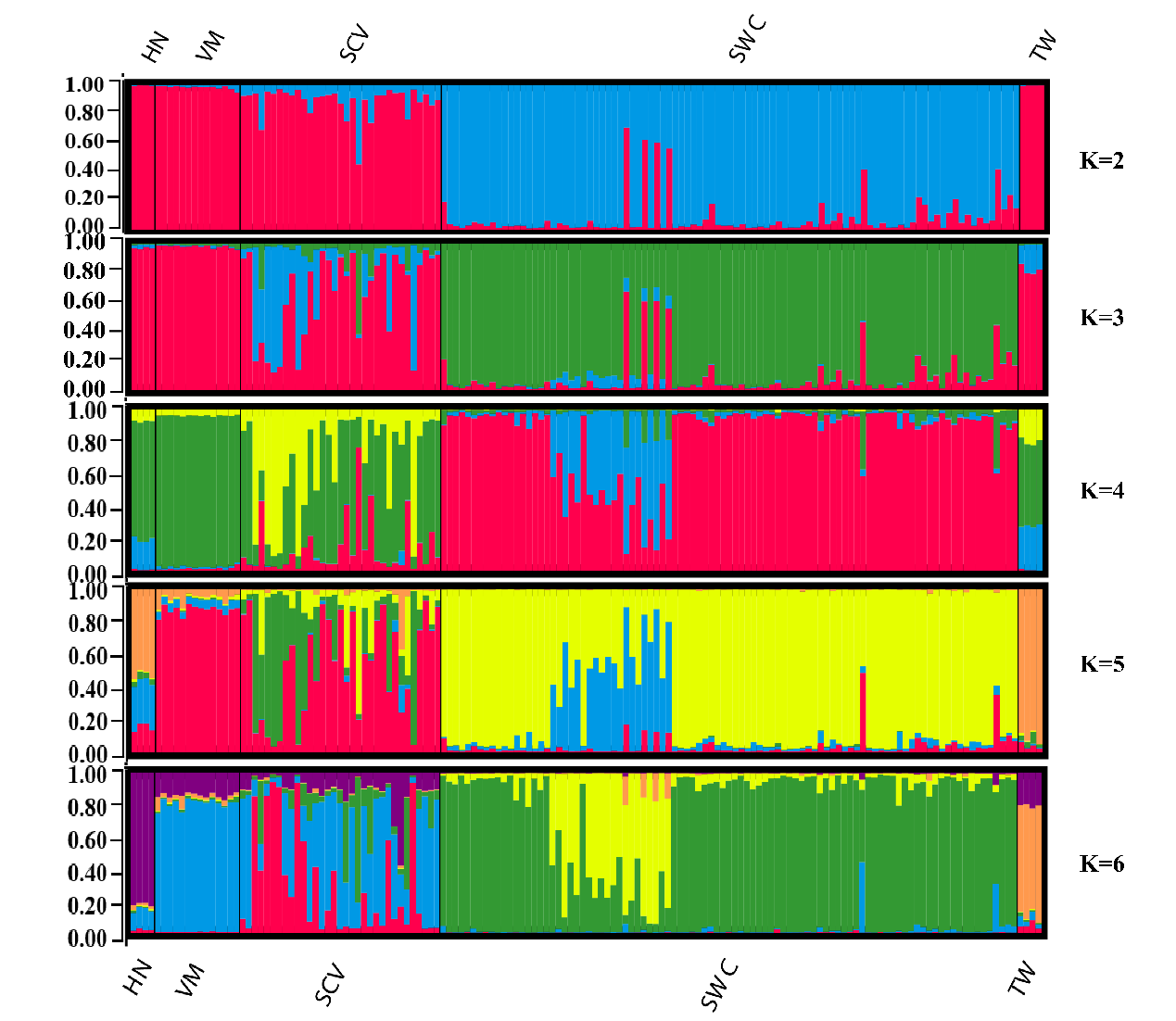

Supplement: Supplementary file 4 — Appendix S4 [file ECE3-12-e8652-s006.docx]
