# Supplementary material for: Genetic diversity and sex‐biased dispersal in the brown spotted pitviper (Protobothrops mucrosquamatus): Evidence from microsatellite markers
Source: Ecol Evol. 2022 Mar 1;12(3):e8652. doi: 10.1002/ece3.8652 (PMC8888261; doi:10.1002/ece3.8652)
Supplement: Supplementary file 5 — Appendix S5 [file ECE3-12-e8652-s004.docx]

**APPENDIX 5**  Analysis of molecular variance (AMOVA) of *P. mucrosquamatus* populations

| Source of Variation | df | Sum of squares | Variance components | Percentage of variation (%) |
| --- | --- | --- | --- | --- |
| Among Populations | 4 | 83.821 | 0.319 | 4% |
| Among Individuals within populations | 145 | 1165.602 | 1.044 | 14% |
| Among Individuals | 150 | 892.500 | 5.950 | 82% |
| Total | 299 | 2141.923 | 7.313 | 100% |
