# Supplementary material for: Genetic diversity and sex‐biased dispersal in the brown spotted pitviper (Protobothrops mucrosquamatus): Evidence from microsatellite markers
Source: Ecol Evol. 2022 Mar 1;12(3):e8652. doi: 10.1002/ece3.8652 (PMC8888261; doi:10.1002/ece3.8652)
Supplement: Supplementary file 6 — Appendix S6 [file ECE3-12-e8652-s005.docx]

**APPENDIX 6**  Pairwise genetic differentiation values (Fst) between populations of the whole *P. mucrosquamatus*

| Pop1 | Pop2 | Fst | Nm | p-value | permutations |
| --- | --- | --- | --- | --- | --- |
| HN | VM | 0.085 | 2.690 | 0.001 | 999 |
| HN | SCV | 0.081 | 2.821 | 0.001 | 999 |
| VM | SCV | 0.017 | 14.300 | 0.001 | 999 |
| HN | SWC | 0.115 | 1.919 | 0.001 | 999 |
| VM | SWC | 0.034 | 7.113 | 0.001 | 999 |
| SCV | SWC | 0.026 | 9.376 | 0.001 | 999 |
| HN | TW | 0.163 | 1.282 | 0.001 | 999 |
| VM | TW | 0.097 | 2.329 | 0.001 | 999 |
| SCV | TW | 0.086 | 2.658 | 0.001 | 999 |
| SWC | TW | 0.119 | 1.851 | 0.001 | 999 |
| Cluster 1 | Cluster 2 | 0.027 | 9.028 | 0.001 | 999 |
